# Informing prostate cancer screening policy makers in the European Union: lessons from cancer screening governance and policymaking

**DOI:** 10.1093/eurpub/ckaf066

**Published:** 2025-06-06

**Authors:** Sarah Collen, Arunah Chandran, Paolo Guglielmetti, Ondřej Májek, Monique J Roobol, Hendrik Van Poppel, Sergio Torres-Rueda

**Affiliations:** Policy Office , European Association of Urology, Arnhem, The Netherlands; International Agency for Research on Cancer/World Health Organization, Lyon, France; Former: European Commission’s Directorate-General for Health and Food Safety (DG SANTE), Luxembourg; National Screening Centre, Institute of Health Information and Statistics of the Czech Republic, Prague, Czech Republic; Institute of Biostatistics and Analyses, Faculty of Medicine, Masaryk University, Brno, Czech Republic; Department of Urology, Erasmus MC Cancer Institute, Erasmus University Medical Center Rotterdam, The Netherlands; Policy Office , European Association of Urology, Arnhem, The Netherlands; Department of Urology, KU Leuven, Leuven, Belgium; London School of Hygiene and Tropical Medicine, London, United Kingdom

## Abstract

Prostate cancer (PCa) poses a significant global health threat, with high incidence and mortality rates. In 2022, the Council of the European Union (EU) updated its screening recommendations, prioritizing PCa screening. This signals a crucial step towards establishing new early detection programmes in EU member states. This study investigates the role of policy makers and governance in cancer screening to inform the development of PCa screening. We had a mixed-method study design. First, a rapid review was conducted on policy making and governance in EU-funded cancer screening initiatives. Second, a focus group discussion reviewed study concepts and methods. Third, a systematic literature review was performed and, fourth, a series of in-depth interviews with actors involved in PCa screening pilots was conducted. Data were analysed thematically and the findings are used to propose 10 recommendations for policy makers. The results of the rapid review and focus group discussion framed the study in the context of existing cancer screening programmes across the EU, and highlighted what already exists in terms of governance tools and methodology. The literature review and in-depth interviews presented key learnings from the literature and real-life settings. These findings are reported using a pre-existing conceptional framework for effective health system governance. The study underscores the critical importance of governance in effective cancer screening programmes. Ten recommendations are proposed, including: defining cancer screening governance, allocating budgets and defining common approaches and key performance indicators for evaluation, establishing methods to enhance citizen participation, and reinforcing network governance.

## Introduction

Prostate cancer (PCa) is a global health concern, affecting over 1.4 million men worldwide every year. Approximately 335 000 new cases are reported annually in the European Union (EU), resulting in over 100 000 deaths each year. PCa is the most diagnosed cancer and the third leading cause of cancer death in European men. PCa's risk is strongly associated with age, with one in 11 men expected to develop the disease by the age of 74 [1].

In December 2022, the Council of the EU adopted the new Recommendation on cancer screening, that replaces the Council Recommendation 2003/878/E, and is introducing PCa, along with lung and gastric cancer, as new screening programmes to be considered by EU member states [2]. Emphasizing a stepwise approach, the Recommendations urge member states to pilot and research the feasibility and effectiveness of PCa screening programmes. Notably, the Recommendations propose a screening algorithm involving prostate-specific antigen (PSA) testing and magnetic resonance imaging (MRI) scanning as recommended in recent literature [3]. This signifies an important milestone at both European and national levels towards the establishment of PCa early detection programmes across the EU.

Despite the Recommendations' focus on ensuring ‘appropriate management’ and ‘quality’ in PCa screening programmes, a knowledge gap exists due to the limited presence of population-based PCa screening programmes, therefore little is currently known on the governance of PCa screening. This is potentially a very significant issue as the screening algorithm proposed involves a multi-step approach including potentially a significant number of healthcare professionals and/or centres. The Recommendation presents an opportunity to leverage insights from other current cancer screening programmes to shape effective PCa screening governance and policies.

### Cancer policy framework

**Figure 1.**
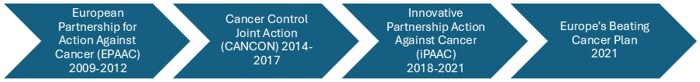
Timeline for EU Joint Actions on Cancer and Europe’s Beating Cancer Plan.

This study takes into account the broader cancer policy framework. For example, National Cancer Control Policies (NCCPs) have been taken into account where possible. Recognized by the World Health Organization (WHO) as a vital policy tool for cancer control, NCCPs guide cancer prevention efforts, even in resource-limited scenarios [4]. The European Commission, through EU-funded Joint Actions on cancer, has actively promoted NCCP development, as depicted in Fig. 1. This is relevant to the current study as these Joint Actions have also developed policy tools on the issue of governance of cancer screening.

The EU Joint Actions and WHO have stressed the need for effective organization of screening, which relies on defined policies and ideally legislation, autonomous management teams, sufficient financing, robust information systems, systematic audits, and periodic evaluations [5]. Governance frameworks and leadership in screening have been emphasized, along with a recommendation to allocate 10%–20% of the programme budget to monitoring and evaluation [6, 7]. The WHO International Agency for Research on Cancer (IARC) and the EU-topia project provide essential criteria and tools for well-organized cancer screening programmes [8, 9].

PCa screening models, which hold promise of reducing screening harms such as overdiagnosis, involve multi-step, risk-stratified screening algorithms, incorporating advanced detection techniques [3]. Good governance is thus crucial in navigating the complexities of this approach. There are ongoing pilot programmes in Swedish regions and a national PCa screening programme in Lithuania [10, 11]. A national pilot programme was also recently started in Czechia [12].

This study draws insights from these experiences to inform policy and address knowledge gaps. The aim of this study is to investigate the roles of policy makers and governance mechanisms in current cancer screening programmes, with the objective of making policy recommendations for policy makers involved in PCa screening pilots in the EU.

## Methods

This study utilized a mixed-methods approach with five components.

### Rapid review

A rapid review of EU-level initiatives on cancer was conducted to map relevant policy makers and governance options deployed in existing population cancer screening programmes. Grey literature from EU Joint Actions on Cancer (EPAAC, CANCON, and iPAAC) was reviewed. The webpages of these Joint Actions were systematically screened for reports containing: definitions of screening governance, policy makers, identified governance challenges, and recommended processes. Information on project partners and actors involved in cancer detection and prevention was also collected. All project reports, policy and training documents, and published papers were reviewed in order to extract relevant information on governance and policy making of cancer screening.

### Focus group discussion

Three policy actors involved in cancer screening policy at the national, EU, and international level participated in the focus group. The discussion aimed to make recommendations on the study design and to highlight key themes that might need to be addressed. A semi-structured interview guide was developed to discuss study aims and methods. The group also reviewed policy actors involved in screening and definitions of governance of screening.

### Systematic literature review

A systematic literature review was performed to comprehend the role of policy makers and governance mechanisms in ensuring the appropriate management of cancer screening programmes. The literature review was conducted using peer-reviewed journal publications and grey literature. Medline, EMBASE, Web of Science, and Global Health were searched using the search concepts of (i) Governance, (ii) Cancer Screening, and (iii) European Union. A full list of all the terms searched, along with a full list of inclusion and exclusion criteria can be found in Supplementary Appendix S1. A grey literature search was also performed to identify NCCPs or screening policies. Articles not focused on the EU and not focused on screening and those published before the first EU Joint Action in 2009 were excluded. Searches of databases were performed on 20 June 2023.

Data extraction was designed around themes found in two existing conceptual frameworks: the EU-Topia Barriers to Effective Screening Tool (BEST) and the TAPIC framework of governance domains impacting health system effectiveness [9, 13]. BEST subsystems represent different phases of the screening process (e.g. generation of knowledge and effectiveness, maximization of uptake), while the TAPIC framework, encompassing transparency, accountability, participation, integrity, and policy capacity (henceforth ‘TAPIC themes’) provide insights into governance process.

### In-depth interviews

Semi-structured interviews were performed with eight policy and operational actors engaged in screening programmes. These stakeholders were chosen among those working in the EU-funded PRAISE-U project, which establishes PCa screening pilots across the EU [14]. The aim of the interviews was to triangulate findings and explore governance aspects in real-life settings, ultimately leading to the development of a set of recommendations. Transcipts of interviews were coded utilizing a deductive thematic analysis approach.

### Formulation of policy recommendations

Policy recommendations were formulated after the literature review and in-depth interviews, using the findings and themes extracted from these study methods. They were shaped in particular on the results of the in-depth interviews.

Ethical approvals were obtained from the LSHTM Ethics Committee and the Ethics Committee of the Katholieke Universiteit Leuven Belgium.

## Results

The results section highlights general findings and explores the TAPIC themes in more detail, reporting the learnings extracted from the rapid review, the focus group, literature review, and interviews.

### General findings

**Figure 2.**
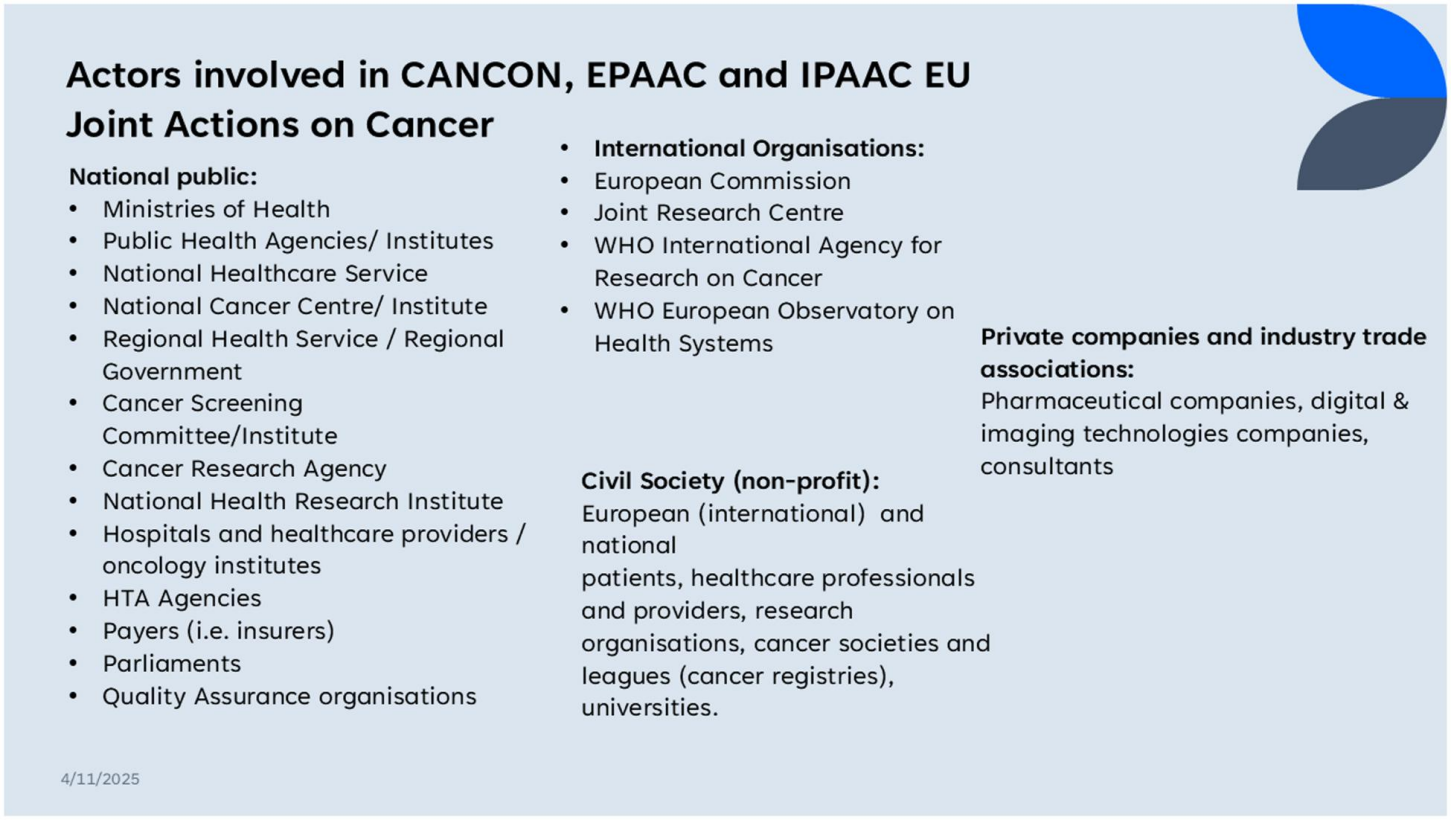
Map of actors identified from EU Joint Action rapid review.

The mapping of actors participating in screening programmes revealed multiple actors involved in the cancer screening pathway, each with different roles, responsibilities, and needs. The actor map is found in Fig. 2.

Definitions of governance in cancer screening were not uniform and thus this study developed a definition of what is meant by cancer screening governance, which was reviewed by the focus group discussion.

The literature review involved an extensive search, resulting in the retrieval of 2361 peer-reviewed articles and 29 records from grey literature. Following the removal of duplicates and application of exclusion criteria, 41 studies and 10 grey literature reports were included in the review.

Table 1 illustrates the PRISMA diagramme and Table 2 illustrates the main findings of the literature review. Table 2 has one axis of the screening pathway taken from the BEST framework, and the other axis uses the TAPIC themes. Table 2 visually demonstrates the number of articles included at each BEST phase and under each governance theme, revealing where there is a concentration of the literature. The identified articles addressed various aspects, including health systems barriers, experiences of cancer screening implementation, challenges in implementing screening programmes, comparisons across the EU, and recommendations for programme evaluation.

**Table 1.** PRISMA 2020 flow diagram for new systematic reviews which included searches of databases, registers and other sources

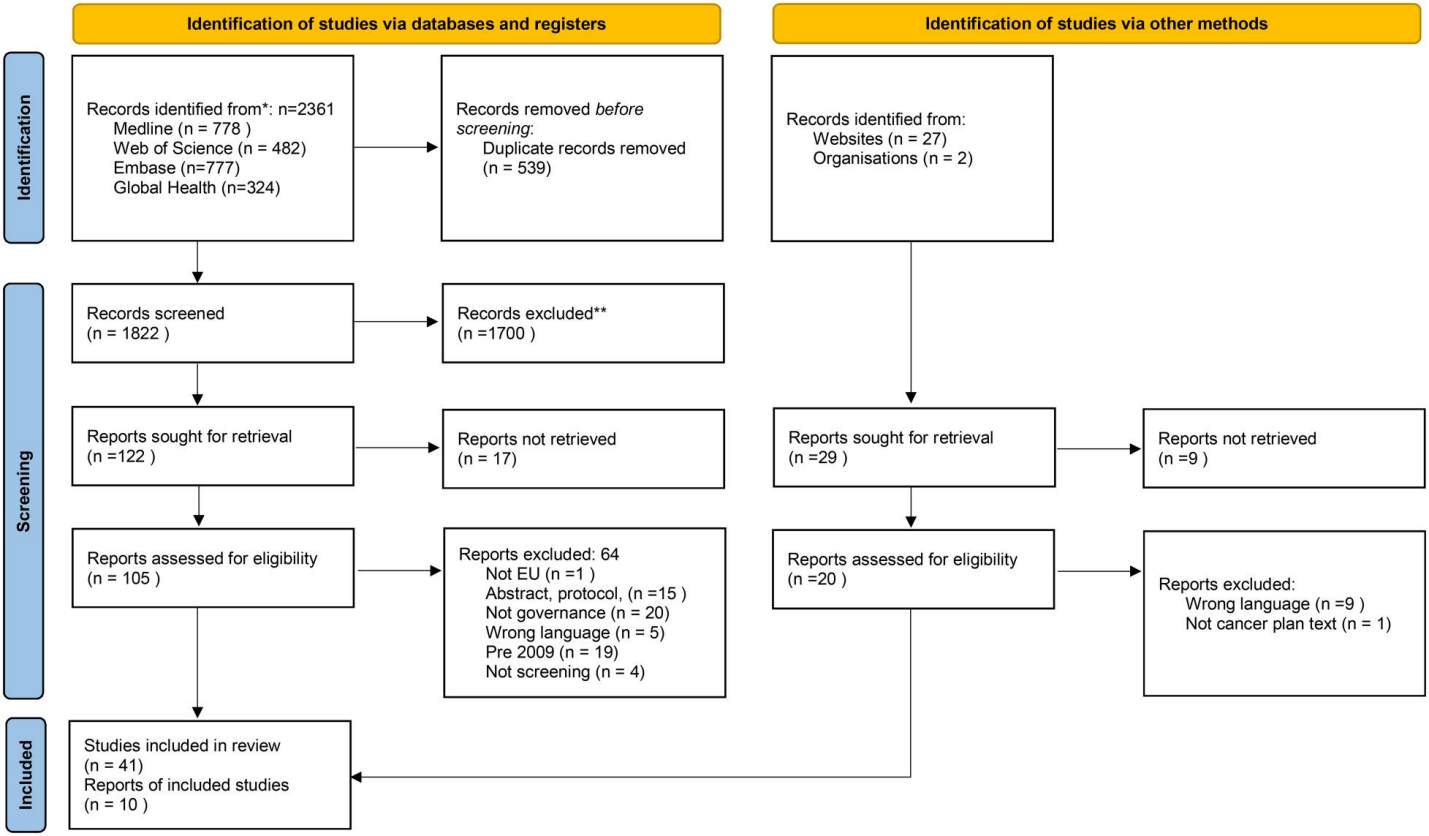

**Table 2.** Heat map of main findings from literature review

	Transparency	Accountability	Participation	Integrity	Policy capacity
Knowledge of effectiveness	12 Articles	12 Articles	13 Articles	12 Articles	36 Articles
Identification of population at risk	4 Articles	No articles	2 Articles	4 Articles	1 Article
Maximization of uptake	1 Article	2 Articles	9 Articles	6 Articles	2 Articles
Operation of the programme	8 Articles	10 Articles	9 Articles	19 Articles	21 Articles
Maximization of follow up	1 Article	No articles	1 Article	6 Articles	1 Article
Assurance of treatment	1 Article	1 Article	No articles	4 Articles	10 Articles

### Results according to TAPIC themes

#### Transparency

The literature emphasized the need for transparent decision-making processes, including the preparation of national legislation on population screening, which can be informed by pilots. Legislation played a vital role in ensuring transparency, and in providing a transparent legal basis for the data linkage that is required for monitoring and oversight of screening.

The importance of a transparent legal base to allow for data linkage was underscored by the interviewee from Czechia:

‘We are now in the category of countries that can actually do these data linkages … it is explicitly […] allowed by the legislation. (Interviewee, Czechia).

Transparency barriers such as confusion arising from fragmentary and overly complex regulations pose additional challenges to screening. Notably, the tension between individual patient rights and population screening requires strong attention to transparency. This is because no screening programme is completely free of risk. According to the interviewee from Ireland, there is considerable uncertainty about false negative screening results linked to a prior incident where some women received inaccurate cervical cancer results [15]. This resulted in successful compensation claims and may contribute to reluctance towards the introduction of an organized PCa screening programme. Individual litigation from those who sadly do not benefit from population screening becomes a barrier and appropriate legal frameworks that transparently acknowledge the risks are required. Additionally, Irish National Screening Service now promotes more transparency in screening decisions [16, 17].

#### Accountability

Accountability was deemed essential, and the literature recommends dedicated teams for screening programme implementation, monitoring, and evaluation [5, 6, 8, 18, 19]. However, challenges were noted particularly during the interviews, including the absence of accountable screening teams. The tension between national and regional decision-making roles was also highlighted.

‘For instance…screening for colorectal cancer was recommended in 2014 and it’s not until…this or next year that all the 21 regions actually have started a screening programme.’ (Interviewee, Sweden).

Quality control and monitoring difficulties for opportunistic screening in private facilities were underlined [9, 20].

‘It's very difficult because when it comes to this unorganized screening, it's about money, the private companies and private labs. They are making money with those tests… And this is also not something… illegal.’ (Interviewee, Poland).

Certification of accredited screening centres and providers can support accountability [21]. In Czechia, the interviewee confirmed that the PCa screening pilot will only permit centres with appropriate staffing, equipment, and training to perform MRIs and prostate biopsies.

‘So this should be hopefully more likely to adhere to guidelines and of course we will also be able to monitor that’ (Interviewee, Czechia).

The importance of having the right incentives in the system to encourage uptake of the agreed screening protocol was deemed important, although it is challenging to implement these in both public and private practice. Examples of this included how to develop best strategies to ensure PSA testing only happens at the recommended time intervals. Also, in terms of maximizing uptake of treatment, that men diagnosed with PCa who are categorized at a phase of low risk of progression of the cancer will be treated with a non-invasive ‘Active Surveillance’ (AS) strategy as opposed to being immediately offered surgery or radiotherapy.

At supra-national level, while it is not possible for international organizations to impose standards or decisions on national governments, international networks that share knowledge and compare data have been created. Accountability at this level thus involves peer pressure between governments.

‘So by…asking Member States to participate in that network, what we do is that we provide a, what we call, “network governance”’. (Interviewee, International organization).

#### Participation

The literature and interviews emphasized the importance of the inclusion of stakeholders, including screening participants, for effective decision-making throughout the screening lifecycle [16–19, 21]. However, the literature revealed a dearth of knowledge on citizen involvement in screening governance. Participatory approaches varied across countries, and the lack of established tools for citizen engagement was noted.

An interviewee from an international health organization mentioned a novel approach to include the views of all stakeholders, called ‘Collaborative User Boards’ which has been tested in cervical cancer screening research, and will be applied in the PRAISE-U pilots. This approach has yet to be published in the literature, but builds on previous research and piloting [22]. Additionally, the steps taken in England to introduce new information on breast cancer screening were cited as an example of good practice of inclusion of views of women [16].

Mechanisms for discussing evidence with key stakeholders before programme approval were present in most countries, but challenges related to lobbying and the neglect of scientific views were identified. According to the interviewee from Lithuania, the Parliament approved a national PCa screening programme because of a strong lobby by patient organizations on the grounds of gender equality. They argued that more must be done for men on cancer screening. This unfortunately neglected the views of scientists and clinicians who did not believe that there was sufficient evidence for the addition of a PCa screening programme at the time.

#### Integrity

This refers broadly to good management where processes, roles, and responsibilities are ethical, clear, and geared to meeting intended aims. Challenges in ensuring the appropriate management of actors and data integrity in screening were discussed in the literature [9, 23, 24]. Processes to ensure data quality and linkage were considered essential for monitoring programme effectiveness [10, 25–30]. The role of cancer registries and screening registries was underscored [5, 6, 31]. The interviews revealed variations in data management processes across countries, highlighting the importance of maintaining data integrity in screening programmes. The existence of evidence based guidelines and standard operating procedures were outlined as essential criteria for screening programmes.

The interviews and literature revealed no agreed common format for audits of cancer screening programmes [16]. However, key performance indicators (KPIs) are being developed under an EU-funded project called Canscreen-ECIS [32, 33]. The PRECEDE-PROCEED barrier audit model has been applied to cancer screening in Italy, but its application has been highly resource intensive and has often only partially been applied [9, 30]. These KPIs must be evaluated over the long term, as it can take significant time to establish if a population screening programme is effective in, e.g. mortality reduction.

In the interviews, no screening programme reported a dedicated budget of 10%–20% of programme budget to monitoring and evaluation advised in the CANCON guide, although all interview participants described it as a component of their programme [6, 31, 34]. Participants of the interviews confirmed the difficulties of securing sustainable funding for these tasks.

#### Policy capacity

This is defined as the ability to develop effective policy that considers available resources to achieve overall goals. All interviewed countries convened local experts to review programmes, develop standards and guidelines, and enhance policy capacity. Piloting was considered a necessary step to ensure a robust programme, and also to build policy capacity to mitigate and respond to challenges [6]. However, there is no agreed international consensus on what criteria pilots should fulfil in order to progress to national roll-out.

EU guidelines, recommendations, internationally recognized performance indicators, and funding were acknowledged as valuable resources for supporting policy capacity and national and international level [8, 35–38]. The need for continuous learning from crises and international collaboration for knowledge sharing was emphasized [16].

In summary, the results encompassed a broad spectrum of findings related to transparency, accountability, participation, integrity, and policy capacity in cancer screening governance. The challenges and recommendations identified provide valuable insights for informing and improving governance mechanisms in cancer screening programmes.

## Discussion

The study reveals a significant body of knowledge across the five TAPIC domains within all screening subsystems of the BEST tool, despite the absence of a clear single definition for cancer screening governance. The TAPIC themes emerged as crucial attributes for effective governance. The interviews and literature review highlighted the necessity of good governance to support policy making, emphasizing challenges across TAPIC domains, and identifying areas of good practice.

The barrier analysis performed using BEST, while not explicitly addressing governance as a theme, lends itself to governance issues. The study anticipates a more targeted focus on governance in the context of PCa screening, particularly due to its risk-based screening algorithm and involvement of multiple actors. The PRAISE-U pilots present an opportunity to address the literature gap and share governance-related experiences, contributing to the development of tools for effective PCa screening governance.

Piloting is crucial in cancer screening implementation. Feasibility studies and pilots serve as training grounds to identify what works and what needs adjustment. The study underscores the lack of international consensus on piloting criteria and suggests the development of agreed-upon criteria for national rollouts, providing valuable guidance for PCa screening policy makers.

The heat map emphasizes Policy Capacity and the Generation of Knowledge of Effectiveness as key areas of focus. Collaborative efforts at the supra-national level have contributed to defining quality guidelines, indicators, and Key Performance Indicators (KPIs). PRAISE-U's role in developing KPIs for PCa screening aligns with the broader goal of enhancing policy capacity and governance across the EU.

The study identifies suboptimal integrity in screening programmes due partly to limited resources for evaluations and audits. The absence of the recommended budget allocation for monitoring and evaluation poses challenges to policy capacity. The literature gap in the accountability domain is highlighted, with discussions on opportunistic, unorganized screening, and payment mechanisms for healthcare providers. European quality-assured accreditation emerges as a potential enabler, addressing accountability challenges in PCa screening.

Data and digitalization are critical enablers for both integrity and policy capacity across BEST subsystems. Barriers to data linkage and use are noted and underutilization of data linkages as a part of screening evaluation was previously reported [29]. The upcoming EU Joint Action on Screening (EUCanScreen) is seen as an opportunity to explore governance and data issues in detail. Allocation of resources for developing data infrastructures aligned with the European Network of Cancer Registries will be vital.

The application of BEST subsystems reveals a dearth of literature in the screening subsystems that link to the follow up of care after initial results and diagnosis. The decline in governance literature in these subsystems suggests a need for further research to address governance aspects during the transition from diagnosis to care. The NCCP is identified as a potential policy tool, emphasizing the importance of cancer plans in governance.

Participatory governance in cancer screening lacks international guidance. Reflection on participatory democracy throughout the full decision-making cycle of screening programmes will be required, suggesting that PRAISE-U's 'Collaborative User Boards' could contribute to addressing this gap.

With the political will from Europe's Beating Cancer Plan, engaging EU countries and the European Commission in the timely development of PCa cancer guidelines is recommended. PRAISE-U can play a crucial role in contributing to the Cancer Screening Recommendation and fine-tuning policy outputs at the European level.

Strengths of the study include combining TAPIC domains and BEST subsystems, offering a conceptual lens for analysing governance in cancer screening. The compatibility of both frameworks allows for a comprehensive analysis of barriers and enablers. In-depth interviews provide valuable insights from those involved in PCa screening implementation. Limitations include the positionality of the PRAISE-U consortium, which may impact views on PCa screening.

Our 10 policy recommendations are included in full below:

1. Cancer screening governance should be defined as a systematic, continuous process of making decisions on screening, and implementing them in health systems to achieve the envisioned outcomes. This usually includes the existence of a NCCP. Attributes of screening governance include the appropriate patterns and mix of transparency, accountability, participation, integrity, and policy capacity.
2. 10%–20% of budget of screening programmes should be allocated for monitoring and evaluation over the long term. Given the learning nature of a pilot, sufficient funding is crucial.
3. The EU-Topia BEST tool effectively identifies barriers and gaps, guiding policy makers towards governance solutions. Given the multi-step, risk-based process piloted in PRAISE-U, the ‘maximization of follow up’ subsystem will require careful reflection to foster accountability. The ‘assurance of treatment’ subsystem should not be overlooked by PCa pilots, thus allowing for preparation of policy options to facilitate Active Surveillance as a treatment option where appropriate.
4. The PRAISE-U network should agree on a standardized approach to PCa programme screening audits to assure quality.
5. Policy makers should define the purpose and scope of pilots, including the criteria that should be fulfilled before moving on to the phase of national roll-out. The PRAISE-U network should allow for sharing of experience on this issue, with a view to development of common agreed criteria.
6. Using the OPT (Swedish regions) indicators as an model, KPIs for PCa Screening programmes should be developed by the PRAISE-U project and integrated into the existing international networks. This would generate possibilities for sharing, learning, and comparing data across countries.
7. There is a critical knowledge gap on guidelines for appropriate involvement of screening participants into the entire governance life cycle of a screening programme. PRAISE-U’s use of ‘Collaborative User Boards’ has the potential to inform this gap by reporting their effectiveness.
8. Findings from PRAISE-U should inform the next periodic report on updating EU Cancer Screening Recommendations, specifically of refining PCa screening approaches.
9. PRAISE-U findings should also inform the development of the future reports to the Commission on the implementation of the Recommendation and of the future EU-level guidelines and quality assurance measures for PCa screening by the Joint Research Centre.
10. Robust evidence based policy for PCa screening hinges on strong data infrastructure. EU funding as well as legal and technical support for cancer screening data infrastructures are crucial.

## Supplementary Material

ckaf066_Supplementary_Data

## Data Availability

The data underlying the interviews in this article cannot be shared publicly due to privacy protection defined in the informed consent.
