# Supplementary material for: Informing prostate cancer screening policy makers in the European Union: lessons from cancer screening governance and policymaking
Source: Eur J Public Health. 2025 Jun 6;35(3):440–6. doi: 10.1093/eurpub/ckaf066 (PMC12198752; doi:10.1093/eurpub/ckaf066)
Supplement: ckaf066_Supplementary_Data [file ckaf066_supplementary_data.docx]

# Appendix A - Search Terms and Inclusion and Exclusion Criteria

Leadership OR Governance OR Stewardship OR Legislat* OR law OR laws OR Policy OR Policies OR Political OR Legal OR Organi*ation OR Strategi* Plan*

AND

Cancer* adj3 (Screen* OR early detect* or cancer* screen*)

AND

European Union OR EU OR E.U. OR Europe OR Spain OR France OR Germany OR Ireland OR OR Sweden OR Finland OR Denmark OR Estonia OR Latvia OR Lithuania OR Czechia OR

Czech Republic OR Netherlands OR Belgium OR Luxembourg OR Malta OR Italy OR Bulgaria OR Romania OR Croatia OR Poland OR Hungary OR Portugal OR Greece OR Austria OR Cyprus OR Slovakia OR Slovenia OR United Kingdom. Although no longer a member of the European Union, the UK was included as there may be relevant literature from the pre-departure period between 2009- 2019. MesH terms were applied in Medline and Embase searches.

MesH were applied in Medline and Embase searches.

A grey literature search was also conducted to find National Cancer policies or screening policies. The following searches were applied in the Google search tool and in the [www.iccp-portal.org](http://www.iccp-portal.org) (each country was searched). Searches on google were made for ‘country name’ + ‘cancer screening’. And ‘county name’ and a ‘cancer control plan’ or ‘cancer plan.’

The inclusion criteria were the following:

- Peer reviewed journal articles;
- Articles focused on implementation of cervix, breast, colorectal and screening programmes in the EU after the start of the ePAAC Joint Action in 2009;
- Articles which describe health systems approaches, barriers and facilitators;
- Articles which explore policy making, governance, leadership, monitoring and evaluation processes for cancer screening;
- National cancer plans or screening frameworks.

*The exclusion criteria were the following:*

- Articles which are older than 2009;
- Articles focused on screening outside EU;
- Articles not on screening;
- Literature that is not focused on breast, colorectal, cervical or PCa;
- Articles that focus on barriers and challenges for individuals as opposed to health systems;
- Articles that do not focus on governance, policy or leadership or monitoring and evaluation;
- Articles that focus on purely clinical policies, management or decision making;
- Articles not in English or French;
- For grey literature, webpage only headline information on screening on cancer control (as opposed to policy documents).
